# Correction to “**Understanding the Interplay of Self‐Regulated Learning Strategies in Medical Education: A Cross‐Sectional Structural Equation Modelling Study**”

**DOI:** 10.1002/hsr2.1769

**Published:** 2023-12-15

Faisal E. Understanding the interplay of self‐regulated learning strategies in medical education: a cross‐sectional structural equation modeling study. Health Sci Rep. 2023;6:e1689. doi:10.1002/hsr2.1689

The acknowledgments for the article is corrected as “This research project was supported by a grant from the Research Center of the College of Education, Deanship of Scientific Research at King Saud University. I want to express my heartfelt gratitude to my husband for his unwavering support throughout this research endeavor. Special thanks are extended to the medical students who willingly participated in this study.”

We apologize for this error.

